# Intra-arterial chemotherapy in locally advanced or recurrent carcinomas of the penis and anal canal: an active treatment modality with curative potential

**DOI:** 10.1054/bjoc.2000.1525

**Published:** 2000-12

**Authors:** A D Roth, C R Berney, S Rohner, A S Allal, P Morel, M-C Marti, M S Aapro, P Alberto

**Affiliations:** Oncosurgery, Department of Surgery, Division of Radio-oncology, Division of Oncology, Geneva University Hospital, 24 Micheli-du-Crest, 1211 Geneva 14, Switzerland

**Keywords:** penile cancer, anal canal cancer, intra-arterial chemotherapy, intra-arterial bleomycin

## Abstract

The prognosis of locally advanced or recurrent carcinomas of the penis (PE) and of the anal canal (AC) after conventional treatment is dismal. We report 16 patients (eight with AC carcinomas and eight with PE cancers) treated by intra-arterial (IA) chemotherapy. Fifteen of them were treated for locally advanced or recurrent disease and one in an adjuvant setting. The chemotherapy was administered via a femoral IA catheter with its tip located above the aortic bifurcation, under the inferior mesenteric artery. It consisted of eight push injections, given over a 48-h period, of the following drug combination: cisplatin 8.5 mg m^–2^, 5-FU 275 mg m^–2^, methotrexate 27.5 mg m^–2^, mitomycin C 1.2 mg m^–2^, and bleomycin 4 mg m^–2^. Leucovorin was given po, 4 × 15 mg day^–1^, during the chemotherapy and for 3 days thereafter. A total of 52 cycles of treatment were administered. Of the 15 patients evaluable for response, six obtained a CR (three PE, three AC) and eight a PR. Among the complete responders, four are alive and disease-free 2–15 years after treatment. The other patients enjoyed an objective response lasting 3–25 months (median 7 months). Four patients developed grade III/IV haematological toxicity with three episodes of febrile neutropenia, one of them with a fatal outcome due to patient's failure to obtain medical attention at the onset of his fever, one a grade III mucositis of the glans, and four a grade III/IV cutaneous toxicity, the latter caused by the IA administration of bleomycin. In conclusion, IA chemotherapy is effective and potentially curative in locoregionally advanced or recurrent carcinomas of the penis and of the anus. Its contribution in the primary management of advanced penile or anal carcinoma should be prospectively investigated. © 2000 Cancer Research Campaign http://www.bjcancer.com

